# Editorial: Overcoming challenges in health technology implementation to maximize patient safety

**DOI:** 10.3389/frhs.2025.1692601

**Published:** 2025-09-25

**Authors:** Melissa T. Baysari, Jane E. Carland, Megan E. Salwei

**Affiliations:** ^1^ Digital Health Human Factors Group, Susan Wakil School of Nursing and Midwifery, Faculty of Medicine and Health, The University of Sydney, Sydney, NSW, Australia; ^2^ Department of Clinical Pharmacology and Toxicology, St Vincent’s Hospital, Sydney, NSW, Australia; ^3^ Center for Research and Innovation in Systems Safety, Department of Anesthesiology, Department of Biomedical Informatics, Vanderbilt University Medical Center, Nashville, TN, United States

**Keywords:** health technologies, patient safety, implemenation, adoption, design, human factors

**Editorial on the Research Topic**
Overcoming challenges in health technology implementation to maximize patient safety benefits

Despite some convincing demonstrations of health information technology (HIT) positively impacting patient safety (1), especially in experimental settings (2, 3), challenges persist in real-world implementation and adoption of these technologies, limiting their expected impact on patient safety. For example, an exploration of how technology was used to support antimicrobial stewardship across multiple hospitals in Sydney, Australia, revealed stark differences between sites in how HIT was used and perceived, resulting in differential impacts of the digital tools on appropriate antimicrobial use (4).

We launched this research topic with a view to collect examples where researchers or organisations had overcome challenges with implementation and adoption of HIT to achieve patient safety benefits. Yet, we were not surprised to receive submissions that primarily described barriers and challenges in their unique healthcare context. In this research topic we heard from authors across a range of countries (Australia, United States, Europe and Africa), who reported on various technologies and contexts. Together, these studies provide additional insights into the many potential benefits to patient safety as well as the challenges of HIT implementation today.

Debono et al.'s study examined nurses' use of workarounds when administering medications in two Australian hospitals using an electronic medication management (EMM) system, a system designed to support medication use (prescribing, administration and review) and minimise medication errors. Observations, interviews and focus groups with nurses revealed that nurses had conflicting feelings about workarounds. Nurses were observed to work around the EMM but described a tension between the perceived necessity of workarounds, and an unwillingness to deviate from policy. The authors highlight that technology-related workarounds can both support and undermine patient safety, and there are clearly times where delivering safe and person-centred care may require nurses to workaround technologies, in ways not intended by technology designers (Debono et al.).

Onyeabor et al.'s study explored the views, obstacles and constraints faced by clinicians during the implementation of electronic health record (EHR) systems across three Nigerian hospitals. Structured interviews with over 300 clinicians revealed the most significant challenges to be those relating to political and managerial commitment, lack of training, and infrastructure limitations (Onyeabor et al.). This study provided important insights on the challenges experienced in developing/resource constrained countries, with healthcare structures and the policy landscape clearly playing a critical role in successful HIT implementation and adoption.

Schubel et al.'s study comprised a workflow analysis to identify optimal implementation strategies for an electronic patient-facing screening tool designed to identify adolescents at risk of a sexually transmitted disease (STI) in the emergency department (ED). Observations in two EDs in the United States, and semi-structured interviews with patients, caregivers, and clinical staff members, identified a range of logistical challenges that would prevent successful implementation, and realisation of benefits from the screening tool. The authors concluded that successful health technologies are those that align with the dynamics of healthcare delivery while also supporting the goals of patients and providers (Schubel et al.).

On a more positive note, Lutz et al.'s study explored the perspectives of healthcare staff on a new platform (IDENTITY), designed to facilitate health information sharing between healthcare staff, child welfare professionals and caregivers of children in foster care. Interviews with healthcare staff working at a paediatric medical centre in the United States revealed that staff were very positive about IDENTITY and in particular the complete patient history it presented (Lutz et al.). Although the technology is in its early stages, this article provides us with a nice example of how technology can offer a potential solution to fragmented information which can lead to sub-optimal care, and can facilitate multi-disciplinary involvement in patient care. The success of this tool appeared to be related to the fact that it addressed a clear and pressing need for multiple stakeholder groups.

Jervelund et al. administered a survey to participants across five countries (*n* = 5,000) to understand factors influencing EU citizens' propensity to obtain prescription medicines online. Results of this large-scale survey showed that only one third of respondents would be likely to order prescription medicines online. Although a number of benefits were reported, including convenience and increased medication adherence, particularly for chronically ill patients, several barriers to adoption were also uncovered. Authors suggest that broad adoption of online prescription medicines in the EU requires a lifting of restrictions on online access, information campaigns to mitigate initial patient concerns, and digital expansion of pharmacies (Jervelund et al.).

In addition to these specific implementation examples, we included one paper that represented a broader contribution to our research topic. Hou et al.'s conceptual analysis paper provided a perspective and model for autonomy support (encouragement for patients to take action on their own) in telehealth, and uncovered two new attributes of autonomy support in this context: technology-based feedback and virtual agent (robots, models, etc which mimic human-like experiences). Looking ahead, the authors propose a roadmap of autonomy support in telehealth which leverages artificial intelligence to adapt to changing needs of patients and thus improve outcomes (Hou et al.).

A take away from the articles in our Research Topic was that challenges with implementation and adoption of health technologies are persistent. Many of the issues uncovered in the papers here were those identified in very early implementations of HIT [e.g., case studies of technology implementations in hospitals over 20 years ago (5)], suggesting we still haven't cracked the implementation puzzle – we are still grappling with the complexities of effective technology design and implementation.

A key theme that emerged from all papers was the importance of context and we recommend future efforts place “context” at the forefront of HIT design and implementation. It is often not clear if “context of use” is sufficiently examined and considered prior to and during the design and implementation phases of a technology. This brings to mind an important question - do we as a community have appropriate methods and approaches to take into account the real environment of use and broader sociotechnical system when designing HIT? The discipline of Human Factors and Ergonomics (HFE) provides us with a range of principles, approaches and methods for technology design and implementation but healthcare has been slow to integrate HFE, lagging behind other safety critical industries in its adoption of HFE (6, 7). Various barriers limit HFE method application in healthcare, including the limited availability of HFE expertise and the complexity and rigidity of available HFE methods (8).

We suggest that for positive impacts of HIT to be achieved, including patient safety impacts, we require a fundamental shift in the way our technologies are designed and implemented, with new methods and approaches that explicitly prioritise context central to achieving benefits. In particular, we advocate for more agile methods that can be applied to understand the context and integrate it into the design and implementation of HIT. We encourage the development of methods that can be applied flexibly, that accommodate how real “work is done” in healthcare contexts (e.g., in teams, across settings), and, given the limited availability of HFE expertise in some healthcare settings, we also call for methods that can be applied successfully by healthcare practitioners and those with variable levels of HFE expertise. As technology gradually permeates every aspect of healthcare delivery across all healthcare contexts, we feel these new approaches and methods are urgently needed and look forward (in anticipation) to future developments in this space.
